# Rapid extraction and analysis of oxidative stress and DNA damage biomarker 8-hydroxy-2′-deoxyguanosine (8-OHdG) in urine: Application to a study with pregnant women

**DOI:** 10.1016/j.ijheh.2023.114175

**Published:** 2023-05

**Authors:** Lucie Bláhová, Tomáš Janoš, Vicente Mustieles, Andrea Rodríguez-Carrillo, Mariana F. Fernández, Luděk Bláha

**Affiliations:** a RECETOX, Faculty of Science, Masaryk University, Kamenice 5, 625 00, Brno, Czech Republic; b Center for Biomedical Research & School of Medicine, University of Granada, Instituto de Investigación Biosanitaria de Granada (ibs.GRANADA), Granada, Spain; c CIBER de Epidemiología y Salud Pública (CIBERESP), Spain

**Keywords:** 8-OHdG, Urine, Oxidative stress, Placenta, Pregnancy

## Abstract

Oxidative stress is an important toxicity and genotoxicity mechanism of many chronic adverse health outcomes. This study developed a sensitive extraction method for urine matrix (based on lyophilization, without the need for pre-cleaning by solid phase extraction), coupled to LC-MS/MS analysis of the biomarker 8-hydroxy-2′-deoxyguanosine (8-OHdG). The methodology was validated in urine samples from a cohort of Spanish pregnant women collected during the first, second and third trimester of pregnancy, and urine samples collected within 24 h after delivery (n = 85). A detection and quantification limit of 0.01 and 0.05 μg/L, respectively, were established. The median 8-OHdG concentration was 2.18 μg/L (range 0.33–7.79); and the corresponding creatinine-adjusted concentrations ranged from 1.04 to 13.12 with median of 4.48 μg 8-OHdG/g creatinine. The concentrations of non-adjusted 8-OHdG significantly decreased (p < 0.05) in the 3rd trimester and post-delivery urine samples when compared to the 1st trimester levels. 8-OHdG concentrations were further studied in placenta samples matching the same urine samples (n = 26), with a median value of 1.3 ng 8-OHdG/g of tissue. Placental 8-OHdG concentrations were correlated with urinary levels of non-adjusted 8-OHdG in the 3rd trimester. Considering the small cohort size, results must be interpreted with caution, however statistical analyses revealed elevated urinary non-adjusted 8-OHdG levels in the 1st trimester of mothers that delivered boys compared to those who delivered girls (p < 0.01). Increased urinary non-adjusted 8-OHdG concentrations at the time of delivery were significantly associated with clinical records (any type of clinical record during pregnancy; p < 0.05). The novel extraction and analytical method for the assessment of 8-OHdG is applicable for sensitive analysis of multiple analytes or biomarkers in urine matrix. This method could also be applied for other matrices such as blood or tissues. Our findings show that 8-OHdG in urine of pregnant women could predict oxidative stress in placenta and can be related to characteristics such as maternal obesity, mode of delivery and newborn sex.

## 1 Introduction

Physiological processes as well as external physical and chemical factors continuously generate reactive oxygen species (ROS). Maintaining balance between ROS production and their removal is an important homeostatic process in all cells. Overproduction of ROS is known to induce pathologies via oxidative damage of important macromolecules and cellular structures (Valavanidis et al., 2009). Biomarkers of oxidative stress have been thoroughly investigated in medicine and toxicology but their importance for environmental health has only recently been postulated (Steffensen et al., 2020).

The hydroxyl radical, the most hazardous ROS, attacks cellular membranes, proteins or nucleic acids including both nuclear and mitochondrial DNA and RNA, where it forms abundant and stable adduct 8-hydroxy-2′-deoxyguanosine (8-oxodG; 8-OHdG) (Halliwell and Gutteridge, 1990; Marrocco et al., 2017). Oxidized DNA can be repaired by various mechanisms and the oxidized adducts are released into urine without further transformation (Fleming et al., 2015). Content of 8-OHdG in urine and plasma is the most studied biomarker of DNA damage, and corresponding health outcomes such as cancer, neurodegenerative disorders and various other chronic diseases (Guo et al., 2017). Levels of 8-OHdG increase with smoking and aging, and with various occupational exposures to physical, chemical, or biological agents (Graille et al., 2020).

The urinary biomarkers of oxidative stress are broadly investigated in preventive or occupational medicine due to several benefits such as non-invasive sampling (Il'yasova et al., 2012; Ventura et al., 2021) or stable matrix less prone to secondary oxidation during sample handling. The biomarker concentrations in urine vary within and between days (Martinez-Moral and Kannan, 2019; Li et al., 2021), and the urinary creatinine is commonly used for normalization although the creatinine excretion rate is also known to be naturally variable (Garde et al., 2004). Levels of 8-OHdG have been investigated in various biomonitoring and epidemiological studies, including also highly vulnerable pregnant women (Zhang et al., 2021), where oxidative disbalance and increased ROS production were documented namely during the 1st trimester (Potdar et al., 2009).

The biomarkers of oxidative DNA damage can be analyzed by using the immunochemical enzyme-linked immunosorbent assays (ELISA) or by selective chromatography techniques with different detection rates (Graille et al., 2020). Because of analytical limitations of ELISA such as inherent lack of specificity (Song et al., 2009; Wu et al., 2004), liquid chromatography-mass spectrometry LC-MS/MS is becoming a preferred and golden standard technique for analyses of oxidative stress biomarkers in biological samples (Chen et al., 2020; Wang et al., 2016).

Although few studies suggested simple dilution or fast ultrafiltration of urine before LC-MS/MS analysis (Qian et al., 2021; Topic, 2014; Zhang et al., 2021), this usually resulted in lower sensitivity (LOQ 0.2–1 ng/mL) due to dilution and signal suppression. Moreover, faster contamination of ion source and shorter column life are also drawbacks of this approach. Correspondingly, pre-treatment of the sample is usually required to remove salts and organic interferences, and to enrich relatively low concentration of some biomarkers. The most commonly used pre-concentration method for urine is solid phase extraction (SPE) (Chen et al., 2020; Guo et al., 2017; Fan et al., 2012; Lu et al., 2016; Ren et al., 2016; Waits et al., 2020; Wang et al., 2016), where many types of sorbents and arrangements are available (Graille et al., 2020). However, multiple steps in SPE are not only laborious but may bring additional variability into the analytical process, and improvements in the extraction and analytical procedures are desirable.

The present study aimed to develop and validate a new and sensitive extraction and analytical protocol for urine samples based on lyophilization, extraction with isopropanol and LC-MS/MS without the need to employ SPE. The performance of the validated method was further demonstrated in a small study with pregnant women by determining the 8-OHdG concentration in several urine samples and the placenta collected at delivery. The correlation between 8-OHdG content in urine and placenta was also investigated, as well as the association between concentrations and data gathered from questionnaires and medical records of participants.

## 2 Materials and methods

### 2.1 Study design

This feasibility study was conducted under the umbrella of the Spanish “Childhood and Environment” (INMA) study. During pregnancy, repeated urine samples were collected, and additional samples (urine and placental tissue) were collected at delivery. For this purpose, 130 pregnant women were recruited at the University Hospital of Granada (Southern Spain), between 2013 and 2015. The basic information related to birth outcomes such as child sex, weeks of gestation, birth weight and child length were collected. All participants were aware of the objectives of the study and provided informed consent to access relevant data from hospital records, including socio-demographic and clinical information. The study followed the principles of the declaration of Helsinki and was approved by the Biomedical Research Ethics Committee of Granada.

### 2.2 Collection of urine and placental samples

Pregnant women (n = 130 in total) provided one urine sample at routine visits to the hospital in each trimester (week 12, 20 and 32 of pregnancy), plus one additional urine sample around the time of delivery (collected during their stay at the hospital). Thus, up to 4 maternal urine samples were collected – the 1st, 2nd, and 3rd trimester and one sample around 24h-post-delivery. All urine samples were collected in polypropylene tubes, aliquoted and immediately stored at −80 °C. The placenta was also collected at the time of delivery, weighted without fetal membranes/maternal decidua, and immediately stored at −80 °C. The frozen collected placentas were then aliquoted into small pieces including maternal and fetal sides as well as central and peripheral parts. In the present work, a subset of samples was used from those mothers who provided both urine samples and placenta. Thus, 26 placental samples and the matching 85 prenatal urine samples were randomly selected from the initial feasibility study, and were sent to Masaryk University, Brno (MU) for analysis. Samples were transported on dry ice and stored at −80 °C until extraction and analysis that was done during summer 2018.

### 2.3 Chemicals and reagents

Standard 8-hydroxy-2′-deoxyguanosine (8-OHdG, 98%) and 2′-deoxyguanosine monohydrate (2 dG, 99–100%) were obtained from Sigma-Aldrich (Merck). Isotopically labelled internal standard 15N5-8-hydroxy-2′-deoxyguanosine (>95%) was obtained from Cambridge Isotope Laboratories. MS grade acetonitrile, isopropanol and formic acid (FA, 99%) were purchased from BIOSOLVE BV (Netherlands).

### 2.4 Urine sample treatment and extraction procedure

For 8-OHdG assessment, the urine samples were thawed at room temperature and homogenized by vortex. The 10 μL of internal standard 15N5-8-OHdG (15N5-8-hydroxy-2′-deoxyguanosine; 1 μg/mL in 0.1% v/v formic acid) was added to 0.5 mL of each urine sample or to calibration solutions of 8-hydroxy-2′-deoxyguanosine (0, 0.05, 0.5, 5, 50 μg/L in 0.1% v/v formic acid) in 2 mL vials. Samples were well vortexed, then gradually frozen at −20 °C and −80 °C overnight and freeze-dried for 24h using freeze-drier (L10-55P, Gregor Instruments). After lyophilization, dry urine and calibration samples were re-suspended in 0.5 mL of isopropanol and extracted in cooled ultrasonic bath for 15 min. Other solvents such as acetonitrile and methanol were also tested for the extraction (data not shown) but the highest recovery was observed for isopropanol. Insoluble material was removed by centrifugation (12 000×*g*, 10 °C, 10 min), 350 μL of supernatants were transferred into a glass vial and evaporated under a stream of nitrogen to dryness (approx. 15 min). Dried extracts were re-dissolved in 250 μL of 0.1% v/v formic acid using cooled ultrasonic bath and vortex. Possible residual particles were removed by centrifugation using microspin filters (0.2 μm; cellulose acetate; Fisher Scientific; 10 000×*g*, 3 min, 10 °C). Filtrates in glass inserts in vials were stored at −20 °C under a nitrogen atmosphere until the analyses of 8-OHdG by LC-MS/MS.

For creatinine analyses, extraction of urine was done according to the previously published study (Dereziński et al., 2016). Analyses used LC-MS/MS method after acidification and dilution of urine in D3-creatitine internal standard solution.

### 2.5 Extraction of placenta for 8-OHdG analysis

Extraction of placenta was based on the previously published methods (Bláhová et al., 2020). Briefly, DNA was extracted from the tissue by using a DNA isolation kit (DNeasy Blood & Tissue Kits, QIAGEN) according to the manufacturer's instructions in two replicates. Enzymatic digestion of isolated DNA was performed by 8-OHdG Assay Preparation Reagent Set (WAKO). To clean the extracts, ultrafiltration (Vivaspin 10 000 MWCO, PES) was used, and the final extract was acidified by formic acid and transferred into glass vials for analyses of 8-OHdG. For analyses of 2 dG, the samples were further diluted 200-fold. All samples were stored at −20 °C under a nitrogen atmosphere until analyses by LC-MS/MS.

### 2.6 Chromatography and mass spectrometry conditions

Analyses of 8-OHdG were performed with Waters Acquity LC chromatograph (Waters, Manchester, U.K.) consisting of a vacuum degasser, a binary pump, a thermostatted autosampler, and a column compartment. The column used was an Acquity UPLC BEH C18 (1.7 μm) (Waters) 100 × 2.1 mm equipped with a guard pre-column kept at 25 °C. Detection was performed on a Xevo TQ-S quadrupole mass spectrometer (Waters Manchester, U.K.) equipped with electrospray ionization. Analytes, after ESI ionization were detected in positive ion mode using tandem mass spectrometry with multiple reaction monitoring (MRM). Data were processed by MassLynxTM software (Manchester, U.K.).

The mobile phase consisted of 0.1% formic acid in water (A) and acetonitrile acidified by 0.1% formic acid (B). The binary pump gradient was linear (5% B at 0–1 min, then increases from 5% B at 1 min to 80% B at 5 min and 80% B was kept for 2 min) followed by 4 min column equilibration to the initial conditions (5% B). The flow rate was 0.2 mL/min, and 10 μL of individual sample from the thermostatted autosampler (10 °C) was injected for the analyses. The ionization parameters were as follows: capillary voltage, 2.5 kV; the source temperature and the desolvation temperature, 150 and 750 °C, respectively; the cone gas flow, 150 (L/h); the cone voltages, 30 V; the desolvation gas flow, 750 (L/h); and the collision gas flow, 0.15 mL/min. The following *m/z* transitions of 8-OHdG were monitored: *m/z* 284.1 > 168.1 (quantifier; collision energy 14V), *m/z* 284.1 > 140.1 (qualifier; collision energy 28V). An average ratio of quantifier (*m/z* 168.1) ions to qualifier (*m/z* 140.1) was stable in standard solution as well as during analyses of urine and placenta extracts and did not exceed 15% of relative standard deviation (RSD).

The *m/z* transition for internal standard 15N5-labled 8-OHdG were also monitored: *m/z* 289.1 > 173.1 (quantifier; collision energy 14V) and *m/z* 289.1 > 145.1 (qualifier; collision energy 28V). For analyses of a parent nucleoside 2′-deoxyguanosine monohydrate (2 dG), extracts of urine were 200-fold diluted, and 2 dG was detected by transition: *m/z* 268.0 > 152.0 (quantifier; 18V) and *m/z* 268.0 > 135.0 (qualifier; 37V). Concentrations of extracted 8-OHdG were corrected for the content of internal standard and expressed as microgram per litre of urine as well as microgram per gram of creatinine. For placenta, the content of 8-OHdG was normalized to the content of 2 dG and expressed in different units to allow for comparison with the literature – i.e. the molar ratio 8-OHdG per 10^5^ 2 dG, ng 8-OHdG per gram of placental tissue and ng 8-OHdG per mg of DNA (Bláhová et al., 2020).

### 2.7 Quality control and data analysis

Quality assurance and quality control samples, including blanks, spiked samples (5.0 ng/mL in 0.1% formic acid) and two urine samples (in house reference material with known lower and higher levels of 8-OHdG) were repeatedly extracted and included in the analysis of samples. Quality control samples (repeatedly extracted samples 5.0 ng/mL in 0.1% formic acid) were analyzed after every 20 urine extracts and found repeatability was acceptable (RSD ≤15%).

Statistical analyses and visualization of the data were performed using Statistica (StatSoft, Inc., Tulsa, OK, USA) and the R programming language, version 4.1.1 (R Core Team, 2021). To achieve a normal distribution and avoid negative values in graphical presentations, the data were log (x+1) transformed using natural logarithm. The paired *t*-test was used to compare urinary mean concentrations of 8-OHdG from different stages of pregnancy. Pearson's correlation coefficient was used to determine correlations between urine and placenta tissue concentrations of 8-OHdG. Due to a small sample size, the non-parametric Mann-Whitney *U* test was used when comparing 8-OHdG in groups with different sociodemographic characteristics or birth-related information.

## 3 Results

### 3.1 Validation of chemical analysis

The novel method used for extraction and analyses by LC-MS/MS in urine samples was validated according to the recommendations of Booth and Simon (2016).

Specificity, i.e. the ability of the method to measure the analyte in the presence of complex matrix, was evaluated by analysing the blanks. No peak was detected at the retention time of 8-OHdG. Quantifier/qualifier ratios (mean ± standard deviation) in different samples were as follows - standard solution 5.1 ± 0.6, urine samples 4.7 ± 0.7, placenta 4.8 ± 0.6. Overall, RSD of ratios did not exceed 15%. The precision of the method was evaluated by carrying out determinations of 8-OHdG replicates in three different urine extracts prepared from in house reference material (no certified reference material is currently commercially available (Topic, 2014)). The coefficient of variation was between 3.9–5.2% and 6.8–9.6% for intra-day (n = 5) and inter-day (n = 5) variability tests, respectively. Data are presented in Supplementary Table S1.

The instrumental limit of detection (LOD) and limit of quantification (LOQ) for 8-OHdG were determined for spiked concentrations in acidified water using a signal to noise ratio criteria (S/N). The limit of detection, 0.01 μg/L, was assessed as the concentration that yielded S/N ratio >3; and the limit of quantification, 0.05 μg/L, as the lowest amount of analyte in the sample that can be quantified, S/N > 10. A precision was further characterized at the LOQ level by injecting a standard of 8-OHdG 6-times (n = 6) into LC-MS/MS. The calculated coefficients of variation were 8.4% for determined concentration and 3.3% for the retention time repeatability**.** Linearity was assessed by repeated analysis of the calibration solutions (four points in two replicates) independently prepared, extracted and analyzed on 3 different days. The coefficients of variance (%) for 8-OHdG of the 3 independent calibration solutions did not exceed 24%. Calibration curve of stock solutions at four concentration levels from LOQ to 50 μg/L (0.05, 0.5, 5, 50 μg/L) were plotted between the responses of peak versus analyte concentrations corrected for responses of internal standard (10 μg/L of 15N5-8-OHdG in each standard concentration). This resulted in r^2^ > 0.999.

The recoveries of the complete extraction and analytical procedure for 8-OHdG were 101 ± 5%, 98 ± 7% and 100 ± 5% for standard solutions of concentrations 0.5, 5 and 50 μg/L, respectively (five replicated experiments, n = 5). Standard solutions were always processed the same way as the urine samples (Supplementary Table S2).

Validation of the recovery was further performed by extracting three different urine samples from healthy volunteers with known concentrations of 8-OHdG (2.13 ± 0.11; 6.35 ± 0.31 and 10.07 ± 0.39 μg/L) after external spiking of two selected 8-OHdG concentrations (5 and 50 μg/L as low and high level of 8-OHdG, respectively, n = 2 for each concentration). The recovery for two spiked concentrations ranged from 84 to 106% (Supplementary Table S3). Lower spike of 8-OHdG (0.5 μg/L) could not be tested because of unavailability of sufficient amount of urine sample with comparable, i.e. low 8-OHdG concentration.

The stability of 8-OHdG in solution of 0.1% FA was determined by analysing reference standard in vials kept at −20 °C under nitrogen atmosphere. Tested solutions were stable for at least 1 month. The example of LC-MS/MS chromatogram obtained in multiple reaction monitoring mode (MRM) for 8-OHdG and 15N5-8-OHdG in urine extract is shown in Supplementary Fig. S1.

### 3.2 Application of the validated extraction and analytical method

**Fig. 1:**
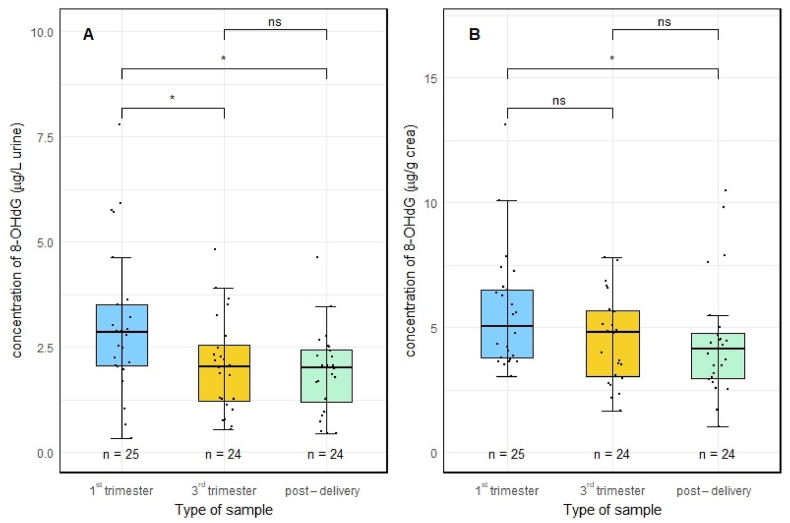
Concentrations of 8-OHdG in urine samples (A - unadjusted urine concentrations, B – creatinine-adjusted concentrations) during the 1st and 3rd trimesters and at time of delivery (ns **-** statistically insignificant; *significant at p ≤ 0.05 by paired *t*-test).

Validation of the new method was performed on urine samples from pregnant women collected throughout their pregnancy. Individual results are presented in Supplementary Table S4. The unadjusted median concentration of 8-OHdG in all urine samples (n = 85) was 2.18 μg/L (range 0.33–7.79). The median of concentration adjusted to creatinine was 4.48 μg/g creatinine (range 1.04–13.12) (Fig. 1). The median urinary concentration of creatinine was 457 mg/L (range 92–1270) (Supplementary Fig. S2). No significant differences in the concentrations of creatinine during the course of pregnancy were observed. The concentration of 8-OHdG significantly decreased (p < 0.05) in post-delivery urine samples (based on both unadjusted and creatinine adjusted values) when compared to urine samples obtain at the beginning of pregnancy (1st trimester). For unadjusted values, 8-OHdG (μg/L of urine) systematically decreased (p < 0.05) during pregnancy, comparing the 1st trimester with the 3rd trimester, and with urine at time of delivery (Fig. 1). Because of the small number of samples available, results for the urine collected during the 2nd trimester were included only in the overall statistics and were not analyzed in detail (Supplementary Table S4).

**Fig. 2:**
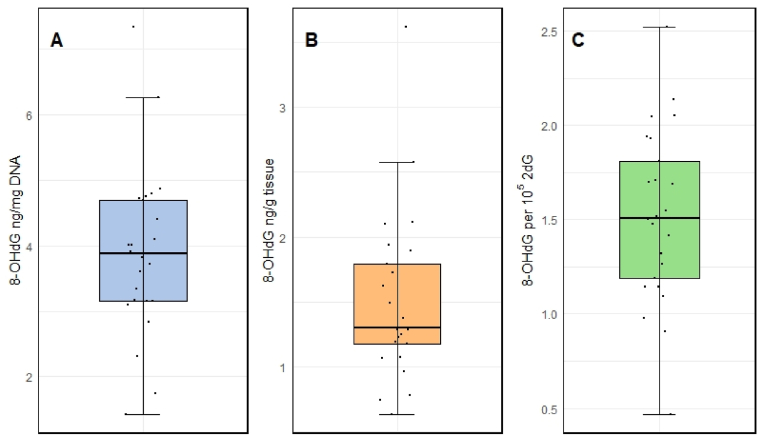
Concentration of 8-OHdG in individual samples of placenta collected at time of delivery. Values expressed in different units – panel A: ng 8-OHdG/mg of DNA, panel B: ng 8-OHdG/g of tissue, panel C: molar ratio of 8-OHdG per 10^5^ 2 dG.

The concentration of 8-OHdG in placental tissue was also assessed, with a median concentration of 3.88 ng/mg of DNA (range: 1.43–7.34), which corresponded to 1.3 ng/g of tissue (0.63–3.62) or ratio of 1.5 8-OHdG per 10^5^ of 2 dG (0.47–2.52) (Fig. 2).

As a next step, we studied correlations between 8-OHdG in urine at different pregnancy stages as well as correlations between urine and placenta concentrations. As expected, the correlations were observed for inter-correlated variables (concentrations in the same samples expressed in different units; results not shown). Interestingly, a significant correlation was observed between unadjusted urine concentration of 8-OHdG (μg/L urine) from the 3rd trimester with the concentration of 8-OHdG in placenta expressed as a ratio per 2 dG (Pearson's correlation coefficient = 0.4, p < 0.05) (Table 1 and Supplementary Fig. 3). The correlations for creatine-adjusted concentrations were not statistically significant.

**Table 1:** Pearson correlation coefficients (r) and p-values among concentrations of 8-OHdG in urine and placenta.

Urine 8-OHdG	Placenta 8-OHdG					
	per 10^5^ 2 dG		ng/mg DNA		ng/g tissue	
	r	p-value	r	p-value	r	p-value
1st trimester (μg/L)	−0.11	0.593	−0.23	0.262	−0.18	0.393
3rd trimester (μg/L)	**0.4**	**0.047**	0.15	0.456	0.22	0.297
post-delivery (μg/L)	−0.17	0.403	−0.18	0.433	−0.17	0.415
1st trimester (μg/g creat.)	−0.1	0.625	−0.24	0.174	−0.03	0.879
3rd trimester (μg/g creat.)	0.38	0.059	0.27	0.176	0.32	0.119
post-delivery (μg/g creat.)	−0.02	0.91	−0.04	0.878	0.02	0.957

Significant correlation (p ≤ 0.05) is highlighted in **bold**.

**Fig. 3:**
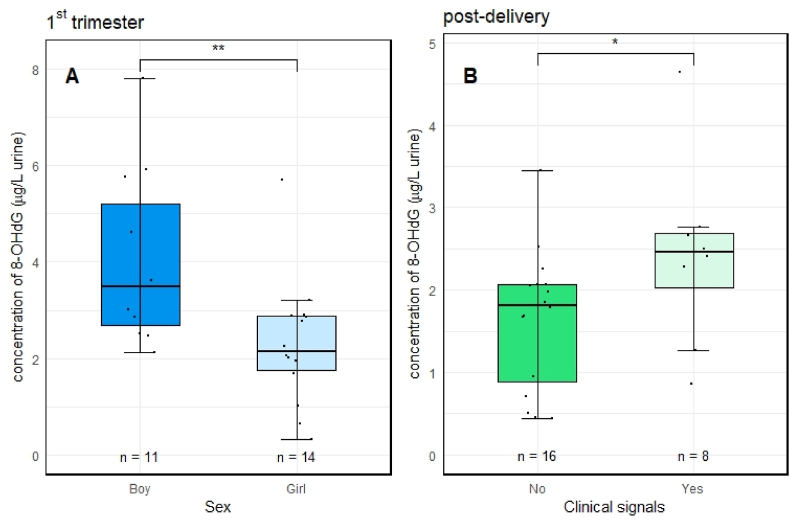
Significant differences in concentrations of 8-OHdG (μg/L urine) between different sub-groups of samples. A – higher 8-OHdG concentrations in the 1st trimester urine at mothers that delivered boys; B – higher 8-OHdG concentrations (urine collected at time of delivery) of mothers with recorded clinical signals (any type of clinical records during pregnancy pooled) (* significant at p ≤ 0.05; ** significant at p ≤ 0.01 by Mann–Whitney *U* test).

Additional statistical analyses were performed to investigate associations between 8-OHdG and parameters from questionnaires and clinical records (placenta weight, head circumference, child sex, mother and child BMI, gestation age, mother age at delivery, tobacco habit, education level; for full data see Supplementary Table S4). Considering a small sample size, the results must be interpreted with caution. Nevertheless, the analysis showed some interesting associations (Fig. 3). Thus, mothers that delivered boys had significantly higher urinary 8-OHdG concentrations in the 1st trimester (p < 0.01). Slightly elevated urinary 8-OHdG concentrations at the time of delivery were associated with clinical signals (Table S4) during pregnancy (any type of clinical disease recorded during pregnancy; p < 0.05). For newborn sex, the differences remained robust (p < 0.001) also when controlling for potential confounding factors (mother's age, BMI, education, tobacco use during pregnancy, parity) (Fig. 3).

## 4 Discussion

The main objective of the present study was to develop and evaluate a simple, sensitive, and reproducible extraction method for determination of 8-OHdG, a widely used oxidative stress biomarker, in urine samples. Various analytical procedures have been previously described including simple dilution of urine samples followed by injection to LC-MS/MS (Al-Saleh et al., 2017; Qian et al., 2021). However, the injection of unprocessed urine has major limitations, namely for larger batches of samples because this approach severely contaminates the ion source in LC-MS/MS, causes signal suppression, leads to a shorter column life and results in insufficiently high detection limits (LOQ 0.2–1 ng/mL; Topic, 2014).

The main broadly used technique for analyte pre-concentration and removing of potential interferences is SPE, most commonly based on reversed phase or cation exchange cartridges (Fan et al., 2012; Potdar et al., 2009; Ravanat et al., 1998; Sabatini et al., 2004). For handling larger numbers of urine samples, automated on-line SPE instruments can be coupled to LC-MS/MS (Hu et al., 2010).

Our extraction method based on lyophilization showed low limits of detection and quantification (0.01 and 0.05 μg/L, respectively), well comparable or lower than limits previously reported for other extraction procedures (Topic, 2014). Actually, the first step, lyophilization, improves stability of urine samples for long-term storage and for easier processing or transport. Interestingly, the extraction methods based on similar principles - such as QuEChERS - are broadly used in environmental analysis of e.g. pesticides in solid matrices such as soils or sediments (Anastassiades et al., 2003). However, to our knowledge, this is the first time that the suitability of the approach is shown for extraction and analysis of human biological liquid material such as lyophilized urine. Our additional studies indicate outstanding performance of the method also for other biomarkers and analytes such nucleotides and nucleosides and their methylated and hydroxylated variants (Janoš et al., 2023), benzotriazoles, benzothiazoles, and various other UV stabilizers, tyrosine derivatives etc. The present study thus suggests general applicability of the new method for many other analytes or biomarkers in urine or blood samples after lyophilization.

This developed method was used in a small cohort study of pregnant women to investigate 8-OHdG levels, their profiles during pregnancy and eventual associations with some determinants. The median concentration of 8-OHdG in all urine samples of participating pregnant women was 2.18 μg/L urine (or 4.48 μg/g of creatinine). These values are in line with some previous studies, where urinary levels of 8-OHdG were analyzed using the same detection technique LC-MS/MS. For example, interquartile range 1.96–3.67 pmol 8-oxodG/μmol creatinine (4.9–9.2 μg/g of creatinine) at 12 weeks of gestation was reported in a study from the United Kingdom (Potdar et al., 2009). Slightly higher concentrations of 8-OHdG with median 9.96 μg/L were observed in a study of pregnant women from Guangzhou, South China (Zhang et al., 2021). Approximately ten times higher median 8-OHdG concentrations were observed in some studies using ELISA methods, which has however been criticized for low specificity (Hu et al., 2004). For example, following values were reported: gravity adjusted median 130 μg/L in U.S. cohort (Ferguson et al., 2015), median 122 μg/L in the study from Northern Puerto Rico (Ferguson et al., 2014), geometric mean 122.6 ng/mL in a study of pregnant women from Boston, USA (Kim et al., 2019) and median in the birth cohort from China 315.2 μg 8-OHdG/g of creatinine (Pan et al., 2022). On the other hand, another study with pregnant women based on ELISA reported levels of 8-OHdG only slightly higher than those observed in the present paper - median 10.99 μg/g of creatinine (Al-Saleh et al., 2013) Similar median value (4.48 μg 8-OHdG/g of creatinine) based on ELISA was reported in a another study with non-smoker women (Lu et al., 2007). As it is apparent, using different techniques for quantification of urinary 8-OHdG makes comparison between cohorts difficult, and harmonization of measurement methodologies in human studies is needed.

Focusing on the differences in urine 8-OHdG in individual trimesters, the concentrations significantly decreased during the pregnancy and in post-delivery urine samples with the highest levels observed in the 1st trimester (Fig. 1). The observed increased production of ROS in urine of pregnant women is in line with other studies (Jauniaux et al., 2000; Myatt and Cui, 2004). It was also shown that the increased oxidative stress in the 1st trimester (measured as 8-OHdG in urine) can be associated with a risk of small gestational age (SGA) (Potdar et al., 2009). However, increasing amount of urination as well as variability of creatinine in different trimesters should be considered when assessing urinary biomarkers in cohorts of pregnant women (Lee et al., 2021).

Higher ROS production during pregnancy can be linked to higher oxygen demand due to increased metabolic rate. The median concentration of 8-OHdG in placenta tissue of participating pregnant women was 1.5 of 8-OHdG/10^5^ 2 dG, which is close to median reported in an older study investigating DNA adducts in placenta - control pregnant women median 0.86 of 8-OHdG per 10^5^ 2 dG (Daube et al., 1997).

The oxidative status of placenta was previously discussed as a marker of complicated pregnancy or adverse child health later in life (Al-Saleh et al., 2013; Dennery, 2010). In agreement, the present study showed higher 8-OHdG in urine of women with reported clinical signs (Fig. 3), and also correlations between 8-OHdG in urine at the end of pregnancy (the 3rd trimester) and 8-OHdG in placenta (Table 1). Overall, this indicate that urine 8-OHdG may be a good non-invasive early warning biomarker of oxidative damage in human tissues (such as placenta).

With regard to sampling of urine, 24h composite urine samples are considered to be the best option but this is not practical and samples are only rarely available in cohort studies (Graille et al., 2020). Correspondingly, it is recommended to adjust concentrations in commonly collected spot samples to creatinine, osmolality or specific gravity as a surrogate for 24-h collection. In the present study, the urinary concentrations of creatinine in pregnant women ranged between 0.1 and 1.3 g/L, which corresponds to values known to be associated with pregnancy (WHO, 1996). No significant changes in concentrations of creatinine were observed between urine collected in different time points (the 1st, 3rd trimester, at time of delivery). As expected, the correlations were observed for inter-correlated variables, i.e. concentrations in the same samples expressed in different units (unadjusted (μg 8-OHdG/L) or creatinine-adjusted μg 8-OHdG/g creatinine). Although some studies with healthy volunteers (e.g. Martinez-Moral and Kannan, 2019) showed that normalization to creatinine might decrease individual variability, we decided to report both unadjusted and adjusted values for 8-OHdG biomarker as it allows to compare the results with different existing studies.

Despite of a small number of participants, our study might indicate some novel associations related to oxidative stress biomarker. While most of the relationships between 8-OHdG and available questionnaire or clinical parameters were non-significant (including for example no effect of tobacco habits), there was an association with newborn sex. Levels of 8-OHdG in urine in 1st trimester were higher in mothers that gave birth to boys (p < 0.01). Additionally, higher urine 8-OHdG concentrations at the time of delivery were observed in mothers with clinical signals (any type of clinical disease recorded during pregnancy; p < 0.05; Fig. 3). These findings are in line with few previous studies that mentioned increase of oxidative stress in placenta in relationship to various characteristics such as maternal obesity, mode of delivery and medications, newborn sex or smoking habits (Burton et al., 2014; Daube et al., 1997; Hung et al., 2011).

There are some limitations of the present study. First, small sample size decreased statistical power, and the associations should be interpreted with caution. Second, reported correlations and/or differences based on parameters from questionnaires and clinical records were significant only in creatinine non-adjusted urinary concentrations. Thus, the influence of urine dilution could not be excluded. Future studies will help to validate the potential predictive power of urinary 8-OHdG concentrations as a non-invasive biomarker of oxidative stress-related diseases in pregnant women.

## 5 Conclusions

A sensitive extraction method for 8-OHdG in urine samples was developed. The method based on lyophilization does not require demanding solid phase extraction pre-cleaning, has a very low limit of detection of 0.01 μg/L, and seems generally applicable for many other analytes or biomarkers in urine or blood samples. The method was successfully applied for 8-OHdG analysis in urine of pregnant women, and the results indicated higher levels of oxidative stress during early pregnancy (1st trimester) with a decrease at the 3rd trimester and post-delivery urine samples. Despite of a small size, which is a major limitation of the present study, statistical analyses revealed elevated 1st trimester urinary 8-OHdG concentrations (non-adjusted) in mothers that delivered boys (compared to those who delivered girls) and increased 8-OHdG concentrations (non-adjusted) in the urine of mothers with clinical records (any type of clinical disease during pregnancy). Our findings further showed that 3rd trimester urinary 8-OHdG concentrations (non-adjusted) may correlate with oxidative stress in placental tissue.
