# Supplementary material for: Rapid extraction and analysis of oxidative stress and DNA damage biomarker 8-hydroxy-2′-deoxyguanosine (8-OHdG) in urine: Application to a study with pregnant women
Source: Int J Hyg Environ Health. 2023 May;250:114175. doi: 10.1016/j.ijheh.2023.114175 (PMC10186372; doi:10.1016/j.ijheh.2023.114175)
Supplement: Multimedia component 1 [file mmc1.docx]

Supplementary material

Rapid extraction and analysis of oxidative stress and DNA damage biomarker 8-hydroxy-2’-deoxyguanosine (8-OHdG) in urine: application to a study with pregnant women

Lucie Bláhová^1^; Tomáš Janoš^1^, Vicente Mustieles^2,3^, Andrea Rodríguez-Carrillo^2,3^, Mariana F. Fernández^2,3^, Luděk Bláha^1^*

^1^RECETOX, Faculty of Science, Masaryk University, Kamenice 5, 625 00 Brno, Czech Republic

^2^ Center for Biomedical Research & School of Medicine, University of Granada & Instituto de Investigación Biosanitaria de Granada (ibs.GRANADA), Granada, Spain;

^3^ CIBER de Epidemiología y Salud Pública (CIBERESP), Spain

* corresponding author: Luděk Bláha; email: ludek.blaha@recetox.muni.cz; phone +420549493194; address: Masaryk University, Faculty of Science, RECETOX, Brno, Czech Republic

**Table S1:** Assessment of intra- and inter-day variability in 8-OH-dG analyses in urine; SD: standard deviation; CV: coefficient of variation; each sample was analysed 5 times within a day, as well as on 5 different days, independently).

|  | Urine sample # 1 | Urine sample # 2 | Urine sample # 3 |
| --- | --- | --- | --- |
| Intra-day variability (within one day) (mean ± SD; N=5), μg/L (CV, %) | 2.13 ± 0.11  (5.16) | 6.35 ± 0.31  (4.87) | 10.07 ± 0.39  (3.85) |
| Inter-day variability (mean±SD; N=5), μg/L (CV, %) | 2.23 ± 0.22  (9.64) | 6.28 ± 0.43  (6.82) | 9.80 ± 0.88  (8.93) |

**Table S2:** Assessment of 8-OHdG recovery from standard solution, extended calibration range 0.05 – 500 µg/L

| Replicate | 0.5 µg/L | 5 µg/L | 50 µg/L |
| --- | --- | --- | --- |
| 1 | 0.55 | 5.38 | 49.07 |
| 2 | 0.49 | 4.75 | 47.17 |
| 3 | 0.51 | 4.45 | 49.91 |
| 4 | 0.49 | 5.08 | 50.36 |
| 5 | 0.49 | 4.89 | 53.32 |
| Mean ± SD (µg/L) | 0.51 ± 0.03 | 4.91 ± 0.35 | 49.77 ± 2.24 |
| Recovery (mean ± SD in %) | 101.2 ± 5.1 | 98.2 ± 7.0 | 99.9 ± 4.5 |

**Table S3:** Assessment of 8-OH-dG recovery from urine samples (nature content of 8-OHdG was subtracted), extended calibration range 0.05 – 500 µg/L

| Replicate | 5 µg/L | 50 µg/L |
| --- | --- | --- |
| # 1 (urine 2.13 µg/L) | 5.3 | 47.6 |
| # 1 (urine 2.13 µg/L) | 4.9 | 49.8 |
| # 2 (urine 6.35 µg/L) | 4.3 | 46.0 |
| # 2 (urine 6.35 µg/L) | 4.5 | 46.2 |
| # 3 (urine 10.07 µg/L) | 4.4 | 43.2 |
| # 3 (urine 10.07 µg/L) | 4.2 | 46.0 |
| Mean ± SD (µg/L) | 4.6 ± 0.4 | 46.5 ± 2.2 |
| Recovery (mean ± SD in %) | 92.0 ± 8.4 | 92.9 ± 4.3 |

**Figure S1:** Example LC-MS/MS chromatogram for urine sample spiked with 15N5-labled 8-OHdG

**Figure S2:** Concentration of creatinine in studied urine samples (mg of creatinine/L urine) during pregnancy and at time of delivery (ns - statistically insignificant, based on both paired t-test, to assess differences between two groups, and repeated measure ANOVA to compare within person variation among all three pregnancy stages.


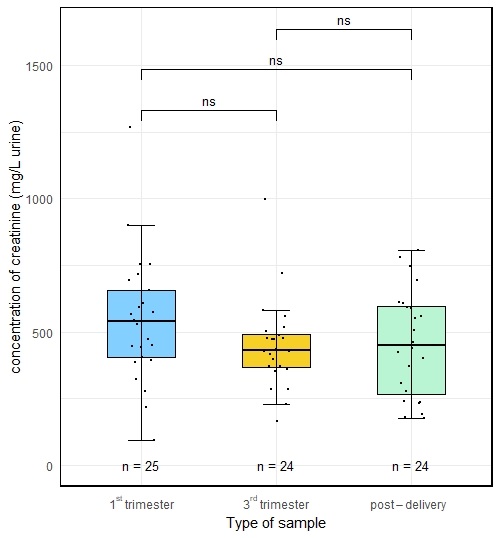


**Figure S3:** Significant correlation between 8-OHdG in placenta (molar ratio of 8-OHdG per 10^5^ of parent nucleoside 2dG) and 8-OHdG in urine (μg/L urine) in the 3^rd^ trimester.


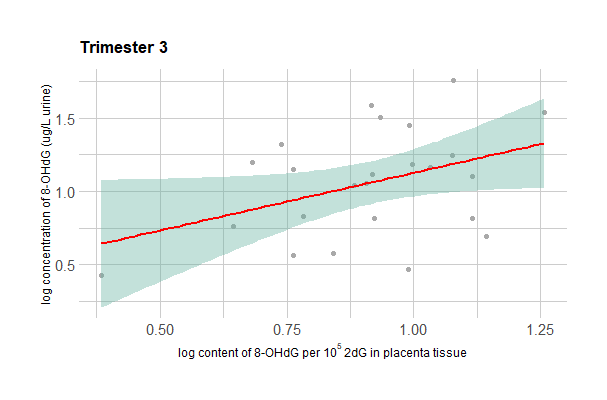


**Table S4:** Concentrations of 8-OHdG in individual urine samples and placentas of the participants and selected socio-demographic and other characteristics of the study participants.

|  | 8-OHdG µg/L of urine | | | | Creatinine mg/L of urine | | | | Placenta | | | Socio-demographic and other characteristics | | | | | | | | | | | | | | | |
| --- | --- | --- | --- | --- | --- | --- | --- | --- | --- | --- | --- | --- | --- | --- | --- | --- | --- | --- | --- | --- | --- | --- | --- | --- | --- | --- | --- |
| Participant | 1st trimester | 2nd trimester | 3rd trimester | post-delivery | 1st trimester | 2nd trimester | 3rd trimester | post-delivery | 8-OHdG/10^5 2dG | 8-OHdG ng/mg DNA | 8-OHdG ng/g tissue | Placenta Weight (g) | Sex | Head Circumference  (cm) | Birth Weight (g) | Birth Length (cm) | BMI | Mother Height (m) | Mother Weight  at 12 weeks (kg) | Mother BMI | Mother Age | Gestational Age  (weeks + days) | Tobacco habit  during pregnancy | Clinical signal  during pregnancy | Educational Level | Previous parity | Delivery year |
| 1 | 1.03 | NA | 1.13 | 2.50 | 276.9 | NA | 370.4 | 552.4 | 0.90 | 2.84 | 1.37 | 498 | Girl | 36 | 3160 | 52 | 11.7 | 1.6 | 56 | 21.9 | 29 | 40+2 | no | Gestational hypothyroidism | University | yes | 2014 |
| 2 | 2.13 | 0.55 | 0.60 | 1.98 | 448.4 | 231.2 | 357.6 | 189.0 | 1.69 | 4.40 | 1.90 | 611 | Boy | 34 | 3560 | 51 | 13.7 | 1.73 | 73.2 | 24.6 | 32 | 39+5 | no | no | Secondary | no | 2015 |
| 3 | 2.91 | NA | 2.05 | 1.85 | 754.7 | NA | 557.4 | 234.8 | 1.50 | 4.09 | 1.29 | 426 | Girl | 33 | 3260 | 49 | 13.6 | 1.6 | 63.7 | 24.9 | 37 | 38+4 | no | no | Primary | no | 2014 |
| 4 | 3.01 | NA | 2.17 | 4.64 | 692.6 | NA | 425.0 | 609.8 | 1.14 | 3.10 | 1.06 | 619 | Boy | 35 | 3370 | 50 | 13.5 | 1.68 | 71.3 | 25.6 | 35 | 39+4 | no | Gestational diabetes | Secondary | yes | 2014 |
| 5 | 4.62 | NA | 4.81 | 2.29 | 1270.0 | NA | 998.3 | 781.1 | 1.94 | 4.01 | 1.62 | 546 | Boy | 38 | 4060 | 56 | 13.0 | 1.65 | 58 | 21.3 | 31 | 40+0 | no | Gestational hypothyroidism | Professional Formation | no | 2014 |
| 6 | 3.50 | 1.09 | 1.29 | 2.06 | 527.1 | 393.4 | 470.8 | 592.9 | 1.19 | 2.31 | 0.63 | 730 | Boy | 35 | 3450 | 52 | 12.8 | 1.64 | 57.5 | 21.4 | 33 | 40+4 | no | no | Secondary | yes | 2014 |
| 7 | 3.21 | 2.18 | 1.00 | 1.78 | 572.9 | 327.8 | 285.5 | 402.1 | 2.14 | 6.27 | 2.11 | 474 | Girl | 33.5 | 2520 | 49 | 10.5 | 1.6 | 62 | 24.2 | 28 | 38+5 | yes | no | Secondary | no | 2015 |
| 8 | 2.25 | 4.29 | 3.26 | 2.67 | 444.8 | 425.7 | 475.7 | 607.9 | 1.70 | 4.01 | 1.30 | 506 | Girl | 35 | 3700 | 53 | 13.2 | 1.64 | 91.2 | 33.9 | 39 | 41+3 | no | Obesity | Professional Formation | no | 2014 |
| 9 | 7.79 | 3.06 | 1.26 | 0.95 | 593.7 | 457.3 | 472.0 | 372.0 | 1.52 | 3.61 | 1.30 | 566 | Boy | 34 | 3410 | 51 | 13.1 | 1.72 | 77.7 | 26.6 | 30 | 37+5 | no | no | Secondary | yes | 2015 |
| 10 | 3.62 | NA | 1.83 | 2.26 | 656.7 | NA | 518.3 | 230.5 | 1.42 | 3.15 | 1.17 | 550 | Boy | 37 | 3530 | 55 | 11.9 | 1.66 | 70 | 25.4 | 33 | 39+5 | no | no | Professional Formation | no | 2014 |
| 11 | 5.70 | NA | 3.91 | 1.69 | 564.3 | NA | 501.6 | 308.2 | 1.50 | 3.90 | 2.10 | 506 | Girl | 34 | 3160 | 49 | 13.2 | 1.63 | 67.2 | 25.3 | 25 | 38+0 | no | no | Primary | no | 2014 |
| 12 | 1.96 | NA | 2.75 | 2.53 | 539.4 | NA | 488.2 | 504.6 | 1.10 | 3.15 | 1.25 | 546 | Girl | 34 | 3450 | 50 | 13.2 | 1.67 | 59.3 | 21.6 | 29 | 40+0 | no | no | Professional Formation | yes | 2014 |
| 13 | 5.76 | 2.79 | 2.32 | 1.67 | 899.9 | 462.8 | 581.9 | 422.1 | 0.98 | 1.43 | 0.74 | 597 | Boy | 33 | 3210 | 50 | 12.8 | 1.69 | 83.3 | 29.2 | 36 | 41+4 | no | no | Primary | no | 2015 |
| 14 | 2.02 | 3.00 | 1.86 | 0.87 | 322.5 | 492.7 | 283.3 | 275.1 | 1.48 | 3.34 | 1.49 | 361 | Girl | 32 | 3160 | 48 | 13.7 | 1.62 | 57.4 | 21.9 | 24 | 39+4 | yes | Gestational hypothyroidism, Infertility (Several abortions) | Secondary | no | 2014 |
| 15 | 2.52 | 1.19 | 2.27 | 2.77 | 718.0 | 467.1 | 395.8 | 589.2 | 1.71 | 4.72 | 1.73 | 597 | Boy | 37 | 3950 | 55 | 13.1 | 1.72 | 76.6 | 25.9 | 28 | 39+3 | no | no | Secondary | no | 2014 |
| 16 | 2.89 | NA | 2.20 | NA | 443.5 | NA | 433.1 | NA | 1.81 | 4.69 | 1.28 | 481 | Girl | 35 | 3510 | 52 | 13.0 | 1.77 | 125 | 39.9 | 29 | 40+6 | no | Otosclerosis, Hypothyroidism | University | yes | 2014 |
| 17 | 2.86 | 2.96 | 0.53 | 2.06 | 394.4 | 422.8 | 228.6 | 560.2 | 0.47 | 1.75 | 0.77 | 457 | Girl | 32.5 | 2760 | 49 | 11.5 | 1.63 | 59.2 | 22.3 | 31 | 38+6 | yes | no | University | yes | 2015 |
| 18 | 2.47 | NA | NA | 2.07 | 607.8 | NA | NA | 462.3 | 1.27 | 4.75 | 1.07 | 855 | Boy | 33.5 | 3110 | 50 | 12.4 | 1.64 | 58 | 21.6 | 30 | 41+2 | no | no | Secondary | yes | 2014 |
| 19 | 2.87 | NA | 2.01 | 0.50 | 387.3 | NA | 413.6 | 179.5 | 2.05 | 3.17 | 1.94 | 606 | Boy | 32.5 | 3400 | 50 | 13.6 | 1.67 | 59 | 21.2 | 32 | 38+5 | no | no | Secondary | no | 2014 |
| 20 | 0.66 | 0.38 | 3.52 | 2.42 | 217.1 | 161.9 | 721.8 | 694.4 | 1.21  (1.88)^a^ | 3.18  (4.25)^a^ | 0.84  (1.08)^a^ | 480 | Girl | 35 | 3320 | 52 | 12.3 | 1.72 | 83.1 | 28.1 | 35 | 41+0 | no | Venous insufficiency | Secondary | no | 2014 |
| 21 | 2.05 | NA | 2.47 | 0.72 | 541.6 | NA | 372.0 | 240.4 | 1.93 | 7.34 | 1.79 | 505 | Girl | 34 | 3090 | 52 | 11.4 | 1.58 | 60.5 | 24.2 | 37 | 40+6 | no | no | Professional Formation | yes | 2014 |
| 22 | 0.33 | NA | 1.26 | 0.44 | 91.6 | NA | 427.2 | 174.7 | 2.05 | 4.86 | 2.58 | 408 | Girl | 31 | 2590 | 47 | 11.7 | 1.59 | 71.5 | 28.3 | 32 | 39+3 | no | no | University | yes | 2014 |
| 23 | 5.91 | NA | 0.76 | 1.27 | 754.3 | NA | 349.8 | 747.2 | 1.14 | 3.82 | 1.19 | 604 | Boy | 35 | 3680 | 50 | 14.7 | 1.65 | 63.5 | 23.3 | 26 | 40+5 | no | Idiopathic thrombocytopenic purpura. Psoriasis. Chronic constipation | University | yes | 2014 |
| 24 | 2.78 | 6.13 | 3.66 | 3.45 | 471.8 | 737.0 | 475.0 | 805.1 | 2.52 | 4.80 | 3.62 | 670 | Girl | 34 | 3440 | 51 | 13.2 | 1.55 | 48 | 20.0 | 27 | 41+4 | yes | No | Professional Formation | yes | 2015 |
| 25 | 1.69 | 0.92 | 0.78 | 0.45 | 403.4 | 239.9 | 162.3 | 437.1 | 1.32 | 3.88 | 1.22 | 580 | Girl | 37 | 3790 | 51 | 14.6 | 1.57 | 50.3 | 20.4 | 26 | 40+5 | no | No | Professional Formation | yes | 2015 |

NA = not available

^a^ two pieces of the placenta were analysed
